# Perceptions of assisted reproductive technologies in wildlife conservation: Public expectations and ethical implications across three EU countries

**DOI:** 10.1371/journal.pone.0342094

**Published:** 2026-02-27

**Authors:** Pierfrancesco Biasetti, Thomas Hildebrandt, Steven Seet, Jan Stejskal, Paolo Giardullo, Frank Göritz, Susanne Holtze, Cesare Galli, Michal Šťastný, Barbara de Mori

**Affiliations:** 1 Department of Reproduction Management, Leibniz Institute for Zoo and Wildlife Research, Berlin, Germany; 2 Ethics Laboratory for Veterinary Medicine, Conservation, and Animal Welfare, University of Padova, Legnaro, Italy; 3 Department of Veterinary Medicine, Freie Universität Berlin, Berlin, Germany; 4 Department of Science Communication, Leibniz Institute for Zoo and Wildlife research, Berlin, Germany; 5 Conservation and Research Fund, Berlin, Germany; 6 Department of Comparative Biomedicine and Food Science, University of Padova, Legnaro, Padova; 7 ZOO Dvůr Králové, Dvůr Králové nad Labem, Czechia; 8 Research Institute for Gene Pool Conservation, Dvůr Králové nad Labem, Czechia; 9 Department of Philosophy, Sociology, Education and Applied Psychology, University di Padova, Padova, Italy; 10 Avantea Labs, Cremona, Italy; Justus Liebig Universitat Giessen, GERMANY

## Abstract

This paper examines public perceptions of Assisted Reproductive Technologies (ART) in wildlife conservation across three European countries: Czechia, Germany, and Italy. In the context of ongoing biodiversity decline, ART can support conservation efforts by promoting population growth, facilitating genetic exchange, and enhancing genetic diversity through genome resource banks. However, the application of ART in wildlife conservation may challenge conventional views on reproduction and extinction and raise ethical considerations, for example concerning animal welfare. To assess public views and ethical concerns, we conducted a survey in three EU countries involved in the BioRescue project, a conservation initiative employing ART to prevent the extinction of the Northern white rhinoceros (Ceratotherium simum cottoni). The survey explored respondents’ attitudes toward biodiversity loss, awareness of the rhinoceros crisis, support for ART in wildlife conservation, and their primary sources of environmental information. The findings inform recommendations for improving conservation communication and fostering public acceptance of ART in wildlife conservation, while emphasizing the need for rigorous ethical oversight to ensure their responsible application.

## 1. Introduction

Assisted Reproductive Technologies (ART) are a set of practices and techniques that involve, in one or more of their stages, the manipulation of reproductive cycles, gametes, or embryos, with the final goal of producing a new individual [1].

In the ongoing biodiversity crisis, the application of ART, when paired with other traditional conservation strategies such as habitat preservation, can make a difference for wildlife populations in rapid decline. ART can be used to overcome inadequate reproductive performance, or to boost the number of individuals produced per generation in conservation breeding programs. They can genetically bridge fragmented populations (living both in situ and ex situ) without the need for translocating animals. Moreover, where genetical variability is low, ART can enable the genetic exchange between living and past generations by using gametes stored in genome resource banks (GRB) [2,3] or, given the possible near-future developments, produced from ovarian tissue or induced pluripotent stem cells [4,5].

It is thus reasonable to predict that the use of ART will become increasingly important and widespread in wildlife conservation [6–11]. For this reason, it is crucial to investigate their public perception and ethical implications in this context [12–17]. Acceptance, interest, and involvement from the public are essential elements for the success of conservation projects [18]. Although many classical types of ART are well established and commonly used in domestic animals, their application in wildlife is still relatively recent, certainly not as widespread and familiar, and may have unique ethical implications. ART redefine our ordinary understanding of what reproduction is and also of when taxa can be considered extinct or seriously threatened [5]. Moreover, they reshape our ideas of what should be the practice and the mission of biodiversity conservation [17], and they may raise significant ethical issues concerning the welfare of the animals involved and the opportunity to use them as a means to ensure the survival of the species [13].

In this paper, we investigate the public perception and ethical implications of several classical ART used in wildlife conservation by presenting and discussing the results of a survey and a media analysis conducted in three European countries—Czechia, Germany, and Italy. These countries were chosen because they host some of the institutions involved in the BioRescue project, an ongoing conservation initiative aimed at preventing the extinction of the Northern White Rhinoceros (*Ceratotherium simum cottoni*, NWR) by using several ART, with the ultimate goal of providing a blueprint for similar ART-based approaches to the conservation of other critically endangered large mammals [2,3,5,19–23]. We cross-referenced these findings with additional data on respondents’ attitudes, beliefs, and commitment to nature and the environment, their awareness of the biodiversity crisis—particularly its impact on the *Rhinocerotidae* family—and the information channels they use to stay informed about these issues. On the basis of this discussion, we propose some general recommendations to facilitate public acceptance of ART in conservation and their ethically responsible implementation.

## 2. Materials and methods

The study was conducted as part of BioRescue, a conservation project aimed at saving the NWR from extinction [5]. There are only two known remaining NWR in the world and both are females incapable of carrying a pregnancy to term. The last chance to avoid final extinction for this taxon is to use biomaterial from both living and dead individuals to produce embryos to be implanted into surrogate mothers of the relatively closely related Southern white rhinoceros [3]. Embryos are currently produced by using oocytes from one of the remaining females and injecting them with cryopreserved sperm from already deceased bulls [5]. To broaden the gene pool, additional embryos will be produced using artificial games to be derived from induced pluripotent stem cells (iPSC) generated from the preserved biomaterial of other deceased individuals [20,21].

This project exemplifies a conservation initiative that employs several forms of ART and may become a blueprint for the conservation of other critically endangered large mammals. In parallel with the development of these new conservation strategies, BioRescue promotes ethical and social reflection on the technologies it implements [15,24–26]. Within this frame, the current study was developed to conduct a bottom-up analysis of potential concerns surrounding emerging strategies for wildlife conservation, using survey data and media analysis. While we do not view public opinion as definitive on these matters, we see a need for a constructive dialogue with society to address concerns and promote acceptance of new conservation strategies.

### 2.1. Ethical statement

This study complied with the relevant European ethical and normative guidelines and was conducted in accordance with GDPR. No specific approval from an ethics committee or institutional review board was required at the time of the study, as participants voluntarily agreed to take part before the beginning of the study.

Participants were recruited from a panel of individuals who had previously consented to voluntary participation in surveys. Before data collection, verbal consent for this specific study was obtained upon contact. Verbal consent was considered adequate because the study involved minimal risk and only required participants to complete an anonymous online questionnaire. No sensitive data or personally identifiable information were collected beyond what was necessary for GDPR-compliant processing. Participants were informed that aggregated results would be used for research purposes and published, and that their data would be processed in accordance with Article 13 of Regulation (EU) 2016/679 (GDPR). They were also informed that clicking the “NEXT” button constituted implicit consent to the processing of their personal data under GDPR, and that they could withdraw at any time or request complete deletion of their data by contacting the designated email address. No waiver of consent was requested or granted.

### 2.2. Definition of ART in the study

ART are usually defined in human medicine as treatments in which male and female gametes or embryos are manipulated in vitro to achieve pregnancy [27]. Veterinary literature generally includes additional procedures, suggesting a broader catalog: for instance, ART can include artificial insemination [28,29], cloning via somatic cell nuclear transfer [6,11], and gamete production from iPSC [5]. For this reason, a suitable definition of ART in wildlife reproduction is any procedure that involves, at one or more stages, the manipulation of reproductive cycles, gametes, or embryos with the aim of producing a new individual.

This definition encompasses a wide range of procedures, some of which have been in use for quite some time—at least in their application to domestic species—while others are more recent. It is therefore useful to distinguish between ART, referring to well-established practices (at least in certain species), and advanced ART (aART), which may require methods involving extensive laboratory equipment and expertise beyond those needed for classical ART approaches [1]. This distinction has a considerable gray area and is likely to shift as once-pioneering procedures become established and are gradually absorbed into classical ART. Nevertheless, it is useful for differentiating, for instance, applications such as artificial insemination on the one hand, and in vitro gametogenesis on the other.

Another important distinction in the discussion of ART in animals concerns the purpose for which these techniques are applied. In livestock and laboratory settings, ART are primarily used to maximize the offspring of genetically desired individuals. Producing large numbers of individuals with recurring genetic traits is, however, neither useful nor desirable in wildlife conservation. In conservation contexts, the goal is instead the establishment or management of populations for reintroduction or for maintaining genetically diverse reserves, with the ultimate aim of conserving the species. This difference in purpose entails different criteria of success and, more importantly, a distinct set of potential implications and concerns, such as the management of genetic diversity or the risk of creating long-term dependencies on human intervention. This means that, even though the techniques may be similar in some cases, and public opinion of ART in conservation may be influenced by familiarity with their application in domestic animals, a specific ethical analysis is still necessary.

Beyond differences in purpose, the use of ART in wildlife conservation also differs from its role, implications and concerns in two adjacent types of practice. The first is synthetic biology, where ART can play an instrumental role as a method for creating new organisms that differ from those occurring in nature or that are shaped by humans through traditional artificial selection, because they possess traits introduced through laboratory genetic techniques. There may be conservation-related reasons for engaging in synthetic biology [30], but these do not exhaust the range of motivations for which these techniques are employed and new organisms constructed.

The second practice is de-extinction. The application of ART to conservation differs from its role in de-extinction techniques in terms both of goals and potential issues. Leaving aside broader concerns about de-extinction projects [31–33], whether a de-extinction initiative falls within the scope of conservation depends on three questions. First, how broad is our concept of conservation—does it, for example, include reconstructing ecosystems, allowing for human and technological intervention? Second, which species is to be revived—does suitable habitat still exist, and can ecological relationships be re-established? Third, which methods are used—can the resulting population be considered, at least to some extent, representative of the species? In this sense, not every de-extinction operation should necessarily be regarded as a form of conservation, although some clearly may be, unless one adopts an extremely narrow definition of the term.

While there may therefore be substantial overlaps in the use of ART across domestic animals, conservation, synthetic biology, and de-extinction, it is useful to keep these applications conceptually separate when analyzing their public perception and ethical implications. In this study, we adopt a definition of ART focused primarily on classical ART (that is, non-aART) as used in wildlife conservation within the framework of existing species. This is the definition to which we refer throughout the remainder of the article when using the term ART.

### 2.3. Survey design and distribution

The research involved three EU countries, Czechia, Germany, and Italy. A draft of the survey was prepared in English and circulated among the members of the BioRescue consortium. A revised version was then finalized by incorporating comments and corrections. This version (see S1 File) was translated by native speaker into the main languages of the three countries involved (Czech, German, Italian).

Recruitment for the survey was carried out in May 2022 through panels provided by a professional agency for each country. Individuals in the panels were invited through the agency to participate until representative quotas for geographical location, age, and gender were fulfilled. Total participants for each national sample reflected the population sizes of the three countries (Czechia: n = 505; Germany: n = 902; Italy: n = 804), with a margin of error of approximately 3%. The samples were then compared with figures from 2022 Eurostat statistics.

This comparison revealed some discrepancies. Middle-aged graduates were underrepresented in the Czech and German samples, while the Italian sample showed an overrepresentation of highly educated individuals over 50. To improve balance across age groups without compromising representativeness, participants aged over 70 and under 20 were excluded from the initial samples. Incomplete responses were also removed. Final sample sizes and percentages, weighted by age group, educational attainment, and gender, are provided in the S2 File.

### 2.4. Survey structure

The survey comprises a demographic section along with eight questions (Q1-8, see S1 File).

(a) *Demographic section*. Demographic data include gender, age, level of education, place of residence, and occupations.
(b) *Environmental concern*. The first three questions examined the respondents’ commitment to environmentalism, their motivations, and their beliefs regarding the ecological crisis.

**Fig 1:**
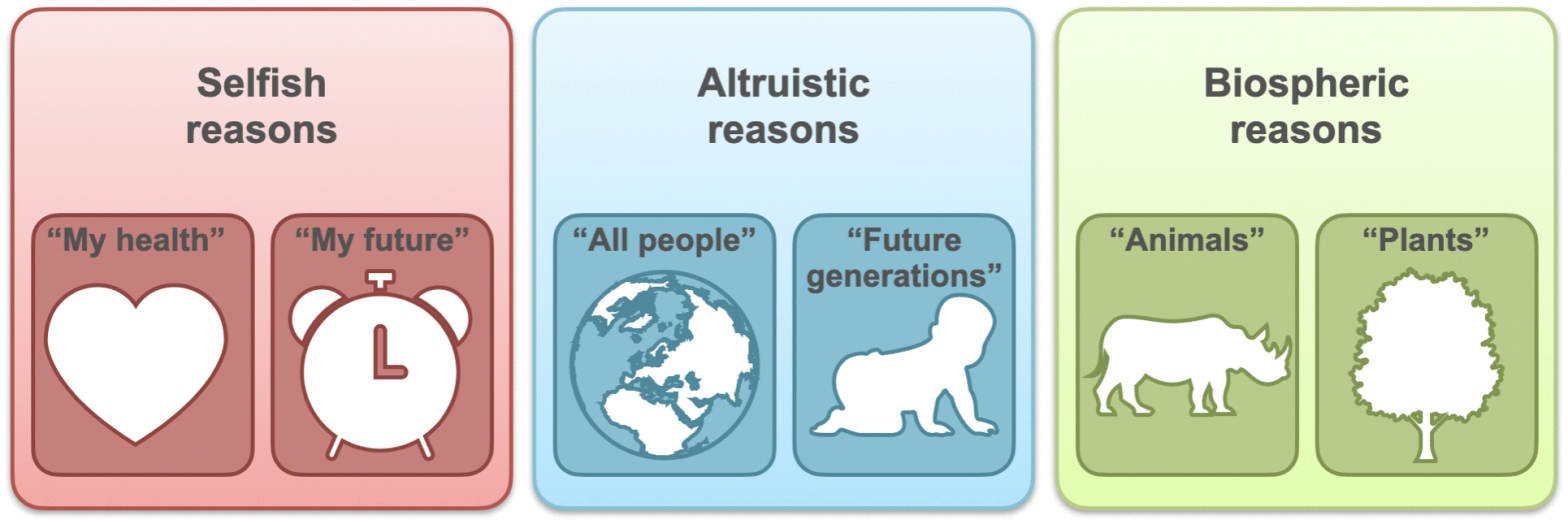
Reasons for environmental concern.

Q1 asked to assign a level of importance (from 1 to 7) to six possible reasons for caring about the environment (**Fig 1**). Each reason is part of a larger category, as identified by Schultz [34]: selfish reasons, altruistic reasons, and biospheric reasons. Two reasons were linked to each factor [35]. To synthesize the results comparatively, we employed the Relative Importance Index (RII)—a composite measure indicating the importance assigned to individual items on a scale from 0 to 1 [36].

Q2 asked respondents how frequently (never, rarely, sometimes, often) over the past year they had engaged in eleven environmentally positive actions. These included action in the private sphere, like recycling or choosing biking over driving, and within the public sphere, such as voting for a pro-environment candidate or signing environmental petitions. These items are being used in large international surveys (Eurobarometer; International Social Survey Programme) and have been validated [37]. Results were used to build an index (0–1) for environmentally friendly behavior.

Q3 investigated the beliefs and opinions of the respondents regarding nature and the environment, asking for their level of agreement (totally agree, mildly agree, slightly disagree, totally disagree, or “I don’t know”) with 11 statements expressing differing views concerning the carrying capacity for humans of the planet, the ongoing ecological crisis, and our place in nature. Results were used to produce three indexes (from 0 to 1) according to a reduced version of the New Environment Paradigma (NEP) scale, which was developed to investigate attitudes and beliefs on the environment [38]. Through Principal Component Analysis it was possible to identify three latent factors: i) Fatalism—the tendency to consider the ecological crisis as unavoidable and unmanageable; ii) Prometheanism—the confidence concerning the possibility of managing the ecological crisis thanks to techno-scientific innovation; iii) Limits to growth—the awareness that humanity has possibly reached the limits of exploitation of the environmental resources.

(c) *Knowledge about the biodiversity crisis*. The next two questions assessed the knowledge of respondents about the biodiversity crisis, with a focus on rhinoceros.

Q4 asked respondents to rate on a scale of 1 (not threatened) to 7 (highly threatened) the extinction risk of six different species of medium to large mammals—three species currently living in Europe, three in Africa. For each continent there was one species assessed by IUCN at the time of the survey as critically endangered (European mink *Mustela lutreola*, black rhinoceros *Diceros bicornis*), one as vulnerable (brown bear *Ursus arctos*, lion *Panthera Leo*), and one as of least concern (European roe deer *Capreolus capreolus*, black wildebeest *Connochaetes gnou*). Both the common and the scientific names were provided. In the survey distributed in Italy, the European mink was replaced by the Mediterranean monk seal (*Monachus monachus*).

Q5 proposed eight possible threat factors to the survival of the extant rhinoceros taxa and asked people to rate the importance of each on a scale of 1 (not a threat) to 7 (extreme threat). The list of threat factors included actual threats (poaching, habitat loss, political and social unrest), non-threats (predators, competition with other species, invasive species), and indirect threats (pollution, natural disasters).

(d) *Attitudes toward ART and possible approaches in conservation*. These two questions investigated the opinion of respondents about ART and, more generally, of different possible approaches to biodiversity conservation.

Q6 asked respondents to rate the acceptability of 10 different items using a scale of 1 (not acceptable) to 7 (acceptable). The content of the items concerned financing research and use of ART for wildlife conservation, the possibility to manipulate individual animals for conservation purposes, attempts to conserve near-extinct taxa, and the need for ethical reflection on ART.

Q7 asked to rate on a scale of 1 (not acceptable) to 7 (acceptable) various biodiversity conservation strategies, including zoos and aquariums, protected areas, conservation breeding and reintroduction, ART, seed banks, botanical gardens, and biobanking.

(e) *Sources of information*. Q8 provided the respondents with a list of eight possible sources of information (television, newspapers, online encyclopedias, magazines, radio and podcasts, books, social networks and blogs, science journals) and asked them to indicate the two most used to obtain information on science and technology. Additional options allowed respondents to specify other sources, indicate they do not seek information on these topics, or answer “I don’t know”.

### 2.5. Media analysis

In communication of science and technology [39], including communication of environmental issues [40–42], media contribute to shape the representations of reality and of social issues [43,44], assigning a different relevance to different topics according to the coverage they offer. For this reason, a media analysis was devised to complement the survey and provide a more comprehensive investigation.

The media analysis was performed by counting articles covering White rhinoceros in selected newspapers of the three countries involved in the survey in timespan ranging from 2012 to 2022. Newspaper archives were queried through the keyword “White Rhinoceros” translated in Czech, German and Italian (see S3 File).

### 2.6. Data analysis

Data obtained through the survey and the media analysis were combined into an analytical path to analyze: i) support for high-tech conservation strategies and the potential impact of national differences and individual variables; ii) opinions on ART used for wildlife conservation, and the variables affecting a high level of support; iii) level of indecision regarding the ongoing biodiversity crisis, as well as the impact of individual variables (such as gender, age, educational attainment, sources of information in science and technology, pro-environmental behavior, and attitudes) that may influence it.

Data were processed using the JASP 0.18.3 statistical package. In stage 1, we analyzed two interrelated variables describing environmental concern (the Schultz scale and the NEP scale), a composite measure of pro-environmental behavior encompassing both private and public actions, and demographic variables. Simultaneously, we examined the importance respondents assigned to ethical considerations surrounding the application of ART to wildlife, as well as their attitudes toward using public funds for biodiversity conservation, particularly for ART-based strategies. These primary findings were then cross-referenced with additional survey data on respondents’ attitudes, beliefs, and information sources. Data from the media analysis provided contextual insights, completing stage 1 of the analytical process.

These mainly descriptive analyses were subsequently combined in stage 2 in a logistical regression model to unveil the influence of the different variables.

## 3. Results

### 3.1. Media coverage

**Fig 2:**
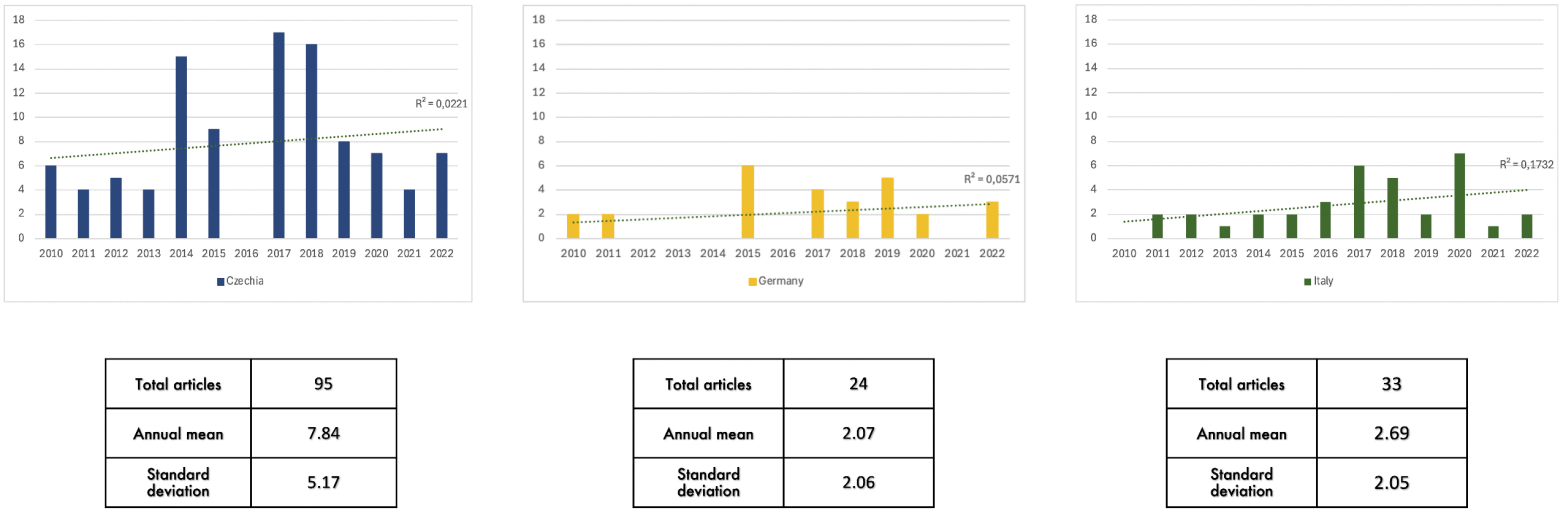
Media coverage of White Rhinoceros (2010-2022).

**Fig 2** displays the number of articles, along with their annual mean and standard deviation, in major newspapers from the three countries that mention the keyword “White Rhinoceros” in their respective national languages.

All countries exhibit a mildly growing trend, with Czech newspapers covering the issue most extensively, averaging 7.84 articles per year—over three times the average for Germany and Italy. Analysis of variance indicates that the average coverage in Czechia is significantly higher than in the other countries (F = 13.935, p = 0.001 for Germany; F = 11.125, p = 0.003 for Italy). In contrast, the difference in average coverage between Germany and Italy is not statistically significant (F = 0.581, p = 0.453). Consequently, we categorized the data into a dummy variable for successive analysis through logistic models: “High coverage” for Czechia and “Low coverage” for Italy and Germany.

### 3.2. Environmental attitudes, beliefs and practices

**Fig 3:**
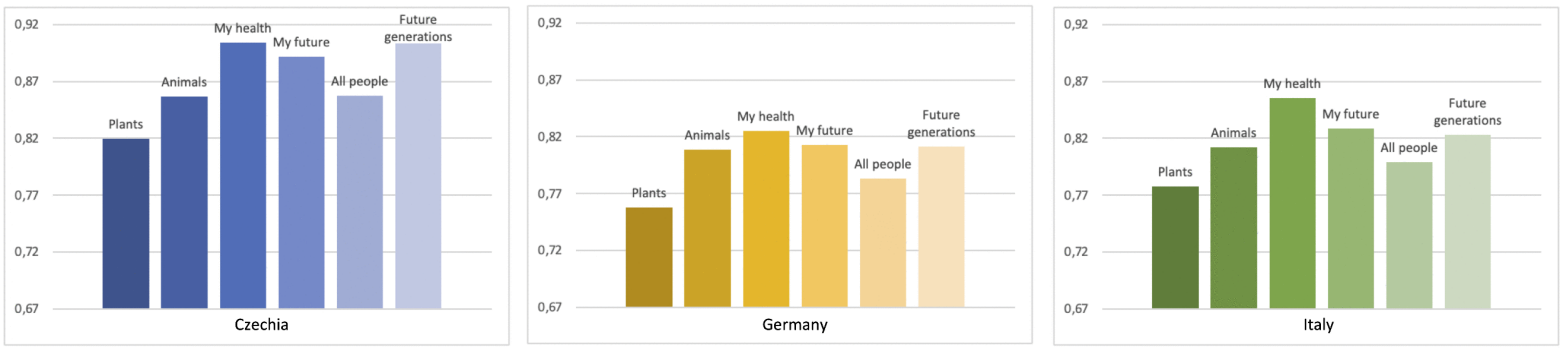
Comparing characterization of environmental concern (RII values).

**Fig 3** presents the results for Q1. Strong environmental concern is evident, with Germany showing the lowest average score (0.800 across six items), while Czechia exhibits significantly higher RII values than the other two countries. Notably, egoistic concerns take precedence over altruistic and biospheric concerns, as respondents prioritize issues related to their personal well-being (“my health”, “my future”). However, the difference between the three motivational categories is minimal. Czech respondents stand out by rating the altruistic concern for “future generations” almost as highly as “my health”. Interestingly, in all countries, concern for “future generations” surpasses concern for “all people”. Biospheric concerns rank lowest across the three countries, with “plants” regarded as less important than other concerns, closely followed by “animals”.

Among those worried about the ecological crisis, gender distribution is not statistically significative, with 49% of individuals scoring 4 or lower on biosphere-related items being male. This figure is notably higher in Czechia (53.24%) compared to Germany (49.07%) and Italy (44.26%).

In Czechia, younger individuals show the greatest concern, with 38.59% under the age of 35, compared to 27.64% in Germany and 26.24% in Italy. Additionally, highly educated respondents (those with tertiary education) are underrepresented in Italy (32.85%) compared to Czechia (60.82%) and Germany (59.42%).

**Table 1** presents the indices for three latent factors (Fatalism, Limits to Growth, Prometheanism) derived from the responses to Q3. These factors characterize respondents’ attitudes toward the ecological crisis.

**Table 1:** Index comparison for environment/society relationship.

	Country	Mean	Std. Dev.	1st Quartile	2nd Quartile (median)	3rd quartile
***Fatalism***	CZE	0.810	0.157	0.750	0.813	0.938
	GER	0.840	0.160	0.750	0.875	1.000
	ITA	0.884	0.128	0.813	0.938	1.000
***Limits to growth***	CZE	0.762	0.203	0.625	0.750	1.000
	GER	0.818	0.187	0.750	0.875	1.000
	ITA	0.798	0.200	0.750	0.875	1.000
***Prometheanism***	CZE	0.581	0.190	0.450	0.550	0.750
	GER	0.575	0.217	0.400	0.550	0.750
	ITA	0.545	0.189	0.400	0.500	0.650

Fatalism emerges as the most prevalent attitude, with its index showing the highest average score across the three countries. The second quartile, reflecting the median value, is also notably close to the index’s maximum. Italian respondents display the strongest inclination toward fatalism, with 59% reporting high or very high levels, compared to 44% in the Czech and German samples. In the Czech sample, the gender distribution skews male, with an 8% higher proportion of males than females. Regarding educational attainment, 31% of Italian respondents inclined toward fatalism had not completed an upper secondary diploma, and Italy has the lowest proportion of respondents with a degree (20.59%) compared to Germany (28.56%) and Czechia (25.21%).

The “Limits to Growth” index indicates that respondents recognize the finite nature of our planet, with German respondents showing the highest mean value for this factor. Across all three countries, individuals with this outlook are typically over 35 and have at least a secondary education. However, in Germany, 32.48% of respondents in this group are under 34, compared to 24.95% in Czechia and 26.85% in Italy. Among those highly aware of this issue, the proportion of respondents with advanced education is notably high. In Germany, this group makes up 30.45%, nearly 9% more than in the other two countries.

Prometheanism scores lower than the other factors in all three countries. Czech respondents show a greater inclination to embrace a Promethean perspective compared to the others, although the median score only slightly exceeds the midpoint of the scale. Analysis of the groups exhibiting the highest levels of Prometheanism reveals a significant gender imbalance, with a predominance of male respondents: 57.85% for Czech respondents, 56.75% for Italians, and 55.11% for Germans. The German group has a higher proportion of highly educated individuals (32.02%) compared to the other two groups (25.97% in Czechia and 20.65% in Italy). Regarding educational attainment, the Czech group predominantly consists of individuals with upper secondary school diplomas (68.68%), a proportion higher than those in the corresponding groups from the other two countries (52.01% in Germany and 49.6% in Italy).

**Table 2** presents the indices related to pro-environmental behaviors, based on answers to Q2. While the combined index shows high similarity across the three countries, the two components—public and private behavior—reveal that people primarily engage in environmental behaviors through personal consumption habits. Public environmental activism is less common.

**Table 2:** Index comparison for pro-environment behavior.

	Country	Mean	Std. Dev.	1st Quartile	2nd Quartile (median)	3rd quartile
**Combined pro-environemnt behaviour**	CZE	0.639	0.161	0.523	0.636	0.750
	GER	0.633	0.149	0.545	0.636	0.727
	ITA	0.672	0.127	0.591	0.682	0.750
**Public pro-environment behaviour**	CZE	0.486	0.219	0.313	0.438	0.625
	GER	0.465	0.221	0.250	0.438	0.625
	ITA	0.468	0.187	0.313	0.438	0.563
**Private pro-environment behaviour**	CZE	0.727	0.167	0.607	0.750	0.857
	GER	0.729	0.162	0.750	0.750	0.857
	ITA	0.789	0.136	0.813	0.821	0.893

Private pro-environmental behavior scores are highest among Italian women (55.55%), while in Germany, women score lower in this regard (43.81%). In Czechia and Germany, individuals with the highest educational attainment are most likely to engage in these behaviors (58.61% and 60.01%, respectively), whereas in Italy, those with medium educational attainment represent the largest share (37.62%). Respondents with low educational attainment exhibit the lowest engagement levels, with 6.08% in Czechia, 12.96% in Germany, and 28.48% in Italy. Across all three countries, respondents are predominantly adults aged 35 and older, making up roughly two-thirds of each sample (Czechia: 64.6%; Germany: 71.91%; Italy: 68.67%).

The number of respondents scoring high in public pro-environmental behavior (index above 0.75) is smaller than those scoring high in private behaviors. Czechia has 74 respondents in this category, Italy 82, and Germany 159. Despite the smaller sample, some trends are evident: similar to private behavior, the majority of individuals with high public pro-environmental scores have a high level of education. In Germany (43.06%) and Czechia, younger age groups are most represented, while in Italy, the age distribution aligns more closely with that of private behavior. The German group skews male (61.51%), while in Czechia, two-thirds are female. The Italian group shows an even gender split.

**Table 3** summarizes the preferred sources of information on science and technology, as explored in Q8. Television clearly emerges as the dominant medium, consistently ranking as the most frequently consulted source across all three national samples. This dominance is particularly notable given the competition from other traditional and digital media, including podcasts, online newspapers, digital encyclopedias, and social media. However, it would be misleading to assume these alternative channels are of lesser importance. Many individuals rely on multiple sources; 72.57% of respondents reported using two, with television most often paired with newspapers (15.53%) or radio and podcasts (13.84%).

**Table 3:** Preferred information sources.

	Czechia	Germany	Italy	Mean
***Television***	72.36%	72.22%	66.51%	70.36%
***Online social media (including video hosting platforms)***	25.32%	18.48%	25.97%	23.26%
***Newspapers***	21.52%	23.88%	24.32%	23.24%
***Online encyclopedias***	21.73%	17.80%	14.81%	18.11%
***Scientific Journals***	10.13%	12.51%	23.50%	15.38%
***Magazines***	10.55%	10.45%	11.63%	10.88%
***Radio & podcasts***	11.18%	12.06%	8.34%	10.53%
***Books***	8.23%	8.27%	8.11%	8.20%
***Other***	0.63%	1.15%	0.00%	0.59%
***Not looking for information***	3.85%	7.34%	3.08%	4.76%

Social media, including platforms like YouTube, Vimeo, and TikTok, are particularly relevant for Italian (25.97%) and Czech respondents (25.32%), but less commonly used by Germans (18.48%). Among social media users, 48% of Italians are under 35, compared to 41.41% in Czechia and 37.41% in Germany. Conversely, the majority of adults over 35 (72.14% in Czechia, 69.4% in Germany, and 73.14% in Italy) primarily rely on television to stay informed about scientific and technological developments. Most of these viewers have medium educational levels; however, in Italy, those with lower educational qualifications make up nearly twice the share seen in the other countries (32.9%). Gender distribution is fairly balanced across the three countries. Another factor to consider is the relatively low proportion of individuals who report not seeking information on scientific and technological topics. This proportion is particularly low in Czechia (3.85%) and Italy (3.08%), and remains modest in Germany (7.34%). The demographic profile of those disinterested in such content is predominantly male, middle-aged, and with lower educational attainment.

### 3.3. Awareness of species at risk, support for ART, and opinions about conservation strategies

**Fig 4:**
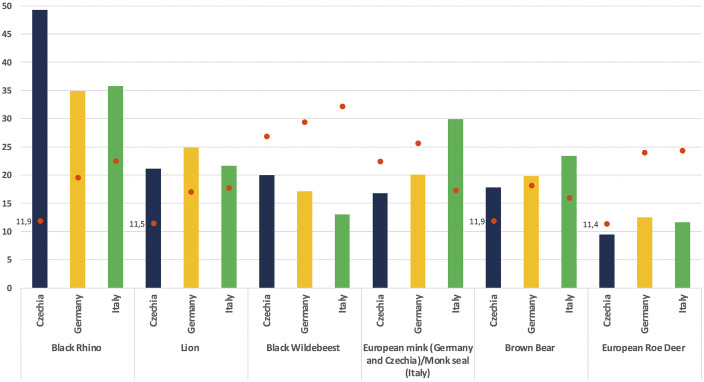
Percentage of respondents estimating as highly endangered (1 of 7 options) the following species (red dots indicate percentage of respondents that opted instead for “Don’t Know”).

**Fig 4** shows the percentage of respondents who classified the species listed in Q4 as “highly endangered” or responded with “I don’t know”. The black rhinoceros received the highest number of “highly endangered” responses across all three samples, particularly in the Czech sample. Nearly half of Czech respondents perceived the species as being at extremely high risk of extinction, with relatively low uncertainty—only 11.9% selected “I don’t know”.

In contrast, uncertainty was notably higher among respondents from Italy and Germany, with approximately one in five expressing uncertainty. Among German respondents, 21.8% of those uncertain were over 55 years old, compared to 14.74% in Italy. The percentage of uncertain individuals with a university degree was also higher in Germany (30.86%), about 10 percentage points above the Italian sample. Gender differences were more pronounced in Germany, where 61% of uncertain respondents identified as female, compared to 57% in Italy.

Regarding respondents who answered “I don’t know”, notable differences emerge among the three country samples. Czech respondents show the lowest overall level of uncertainty in their assessments. Overall, those expressing uncertainty about at least one species tend to be older, with 74.86% over the age of 35. This proportion is highest in Germany (81.41%) and lowest in Italy (68.63%).

Education level significantly influences uncertainty, particularly in the Italian and German samples but not in Czechia. Among uncertain Italian respondents, 30% have a low level of education, compared to 25.6% in Germany and just 6.5% in Czechia. Gender distribution across the samples remains relatively balanced.

**Fig 5:**
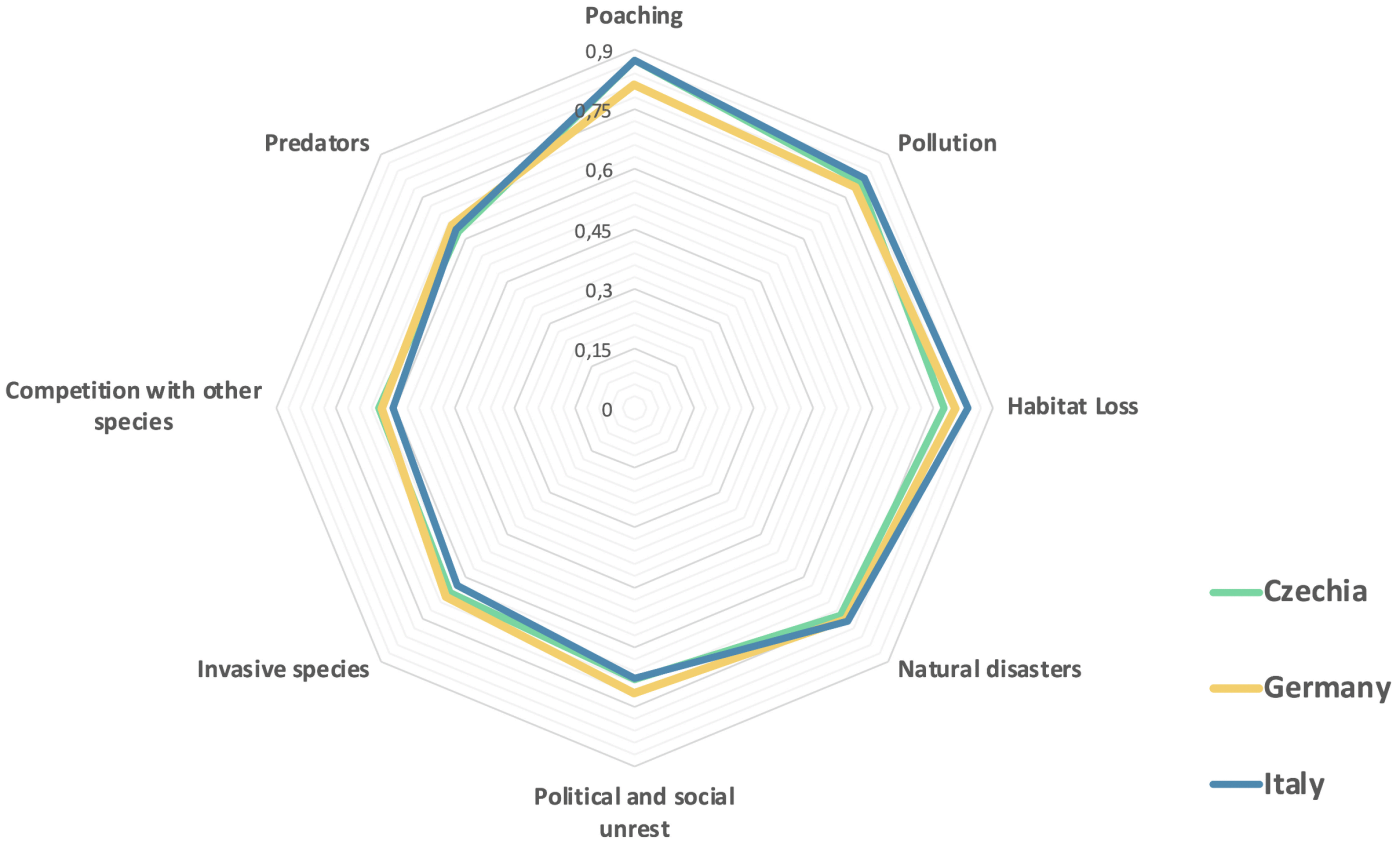
Perceived threats to rhinoceros (RII values).

**Fig 5** presents responses to Q5, showing that respondents correctly identify poaching as the primary threat to rhinoceros. Habitat loss is ranked as the second major threat in Germany and Italy but appears third in Czechia, where respondents overestimate the impact of an indirect factor like pollution. In all three countries, the significance of political and social unrest in contributing to rhino population decline is underestimated.

**Table 4** reports the results of Q7. They highlight a clear preference for the creation of protected areas as the favorite strategy for conservation. Establishing botanical gardens is the second most preferred strategy, while zoos and aquariums are less popular, especially among Italian respondents (but not among Czechs). Generally, new high-tech strategies like ART and GRB are perceived somewhat less favorably than traditional approaches such as protected areas and botanical gardens. Among the three national samples, Czech respondents show the most favorable attitudes toward innovative conservation strategies.

**Table 4:** Acceptability levels for conservation strategies (RII values).

General categories	Components	Items	Czechia	Germany	Italy	Mean
***Traditional (low technology)***	Conservation and protection of plant species and environmental heritage	1. Creation of protected areas	0,867	0,832	0,874	0,858
		2. Botanical gardens	0,823	0,777	0,845	0,815
	Conservation and protection of animal species	3. Zoos and aquariums	0,767	0,675	0,615	0,686
		4. Captive breeding and reintroduction	0,743	0,714	0,733	0,730
***Innovative (high technology)***	Conservation of flora and fauna through new technologies	5. Assisted reproduction technologies (ART)	0,741	0,715	0,736	0,731
		6. Seed banks	0,769	0,714	0,739	0,741
		7. Biobanking (GRB)	0,759	0,674	0,746	0,727

**Table 5** presents the results for Q6, revealing a degree of consensus across the three samples regarding the necessity of investing in ART research to conserve endangered species (item 1). However, consistent with previous findings, protection of natural habitats remains the preferred conservation strategy (item 10).

**Table 5:** Views on ART (RII values).

		Czechia	Germany	Italy
1	Governments should invest public money to support research into advanced assisted reproductive technologies for conserving species extinct in nature	0,747	0,748	0,735
2	Saving a single animal of a species is a first step toward the conservation of the species itself	0,774	0,698	0,399
3	Conservation of almost extinct species is pointless	0,418	0,756	0,741
4	Research groups applying advanced assisted reproductive technologies to protect wild species from extinction should be ethically regulated by the government that funds them	0,677	0,728	0,748
5	We should not push to the point of putting at risk the individual animals, even in trying to save a species	0,727	0,670	0,779
6	Individual animals can be means to reverse the extinction of their species	0,740	0,745	0,748
7	Advanced assisted reproductive technologies should be funded to attempt to preserve almost extinct species	0,755	0,692	0,779
8	Research groups into advanced assisted reproductive technologies for conservation of biodiversity funded by the government should carry out their research activities freely with no ethical constraints	0,655	0,465	0,613
9	Governments should invest public money to support projects which aim to protect endangered species or sub-species rather than those already extinct in nature	0,754	0,703	0,760
10	Governments should support projects which aim to protect natural habitats and limit pollution rather than using advanced reproductive technologies for conserving biodiversity	0,758	0,712	0,807

The three samples differ in their views on the strategic use of ART in wildlife conservation. Czech respondents express more confidence in ART’s potential to save individual animals and, by extension, entire species, compared to Germans and Italians (items 2, 3). Conversely, German respondents emphasize the need for ART in wildlife conservation to be accompanied by ethical reflection on its implications (items 4, 8).

An index of support for ART was calculated based on items 1, 7, and 10 (this latter with a reversed orientation). The average scores for the three national samples are quite similar: Czechia scored 0.629, Germany 0.624, and Italy 0.617. There is minimal variation in score distribution, with a standard deviation of 0.11 for Czechia and 0.12 for Italy. This suggests that the three national samples share a comparable orientation towards ART. However, the highest quartile for the ART index in Germany consists of a younger demographic, with 36.02% of respondents under 35, compared to approximately 25% in Italy and Czechia. The German respondents who support ART the most are also the most highly educated, with 31% holding a degree, while the majority of respondents in Czechia and Italy have only completed secondary education.

### 3.4. Logistic models: what factors have the highest influence?

We constructed a binomial logistic regression model to examine the influence of various factors on: a) support for ART, b) endorsement of innovative biodiversity conservation strategies, and c) uncertainty in estimating species’ threatened status. The model includes individual variables such as Fatalism, Limits to Growth, and Prometheanism, along with demographic factors like age, educational attainment, and gender. Media coverage and country of origin were also considered to account for contextual influences. We first applied the model to the total sample, then repeated the analysis on the three national samples, excluding contextual variables, as these are inherent to each sample. This approach allowed us to assess the consistency of results across the three countries and interpret any potential differences (see S4 File).

The first model shows that a higher level of biospheric concern, as measured by the Schulz scale, is positively associated with support for ART (Odds ratio—OR=3.925, p < 0.001). The same applies to Fatalism and Limits to Growth. Being of median age (35–44 years) also has a notable influence, though less significant compared to attitudes. The non-significance of gender is instead confirmed.

Contextual variables have a different effect. Media coverage (low in Italy and Germany, high in Czechia) does not appear to be relevant, residence in Germany has a protective effect (OR=0.666, p < 0.007); in other words, living in Germany reduces the likelihood of supporting ART. Moreover, biospheric concern plays a predominant role across all three countries—higher for Germany (OR=5.962) and Czechia (OR=3.498) and lower for Italy (OR= 2.678).

The second model assesses the influence of variables on respondents’ support for technological strategies in biodiversity conservation. While biospheric concern remains relevant—suggesting heightened sensitivity toward the ecological crisis among supporters of high-tech strategies—Prometheanism emerges here as the most significant variable. Individuals who are more inclined to favor technological solutions for biodiversity protection have a more than 90% greater likelihood of support for ART (OR=1.909, p = 0.002).

The third model investigates the factors leading individuals to feel more uncertain about the level of threat faced by the species listed in Q4. The importance of Prometheanism is clear, as it appears to significantly promote indecision in estimating the level of risk for various animal species (OR=1.727).

In this model, gender and age play an important role. Respondents in the 55−64 age group are less inclined to express doubt compared to younger individuals (OR=−4.256). This tendency is also observed in the preceding age group, which, while narrowly missing statistical significance, shows a similar trend (OR=−3.122). This suggests that age acts as a protective factor against expressing doubt or uncertainty, more so than educational level.

Once again, contextual factors are not significant. However, examining the national samples individually in Model 3 reveals interesting findings, especially concerning the role of Prometheanism: it significantly contributes to doubt or uncertainty among German respondents, whereas it is not significant for Czech respondents.

## 4. Discussion

The application of ART to wildlife conservation occurs within a framework—the protection of species threatened with extinction—that is already complex from a decision-making perspective. It requires consideration and balancing of the potentially conflicting demands of various stakeholders [45]. Consequently, it is essential that these new conservation strategies undergo rigorous examination through both top-down and bottom-up analytical approaches to address potential ethical concerns and enhance public acceptance.

To our knowledge, this article presents the first investigation into public opinion on the use of ART in wildlife conservation and its ethical implications. It lays the groundwork for a bottom-up analysis of the ethical and social implications associated with these biotechnologies. Bottom-up approaches allow us to identify and prioritize the concerns of stakeholders—in this case, the public—while examining the factors that shape their perceptions. This complements top-down analyses, which identify and debate issues independently of stakeholder views.

The results highlight several topics for further discussion, such as public awareness of the ecological crisis, and, more specifically, of the rhinoceros crisis, the role of media in shaping knowledge about these issues, and the relationship between traditional and new conservation strategies. The findings also provide insight into public support for ART as a conservation approach and underscore the need for ethical considerations to guide the application of these particular biotechnologies.

### 4.1. Awareness of the ecological crisis

Across the three national samples examined, there is a notable level of environmental concern and a fair awareness of the limits of our planet’s resources. As the current rate of biodiversity loss surpasses the critical threshold necessary to maintain the favorable environmental conditions humans have experienced since the Holocene [46] and, according to numerous estimates, aligns with those of past mass extinction events [47], it is essential to assess whether the environmental concern shown by the respondents is matched by genuine knowledge of the ongoing biodiversity crisis.

Our research indicates that increasing age, education level, and Prometheanism are key factors contributing to some uncertainty in estimating the threat levels of species. Additionally, the results suggest a bias in perceived threat, with respondents tending to view exotic species as more endangered than local ones. For example, in the Czech sample, more respondents to Q4 categorized all three African species—including the least threatened, the black wildebeest—as severely endangered, while comparatively fewer recognized the critically endangered status of the European mink. Moreover, across all samples, a higher proportion of respondents correctly identified the black rhinoceros as critically endangered compared to those who recognized the critically endangered status of the European mink or the monk seal. These findings underscore the need for improved communication regarding the threat levels facing local species, which are often underestimated or less widely known.

### 4.2. Awareness of the rhino crisis

Rhinoceros are among the most critically endangered mega-vertebrates in the world, with three of the five extant species classified as “critically endangered” by the IUCN: the black rhinoceros, the Javan rhinoceros (*Rhinoceros sondaicus*), and the Sumatran rhinoceros (*Dicerorhinus sumatrensis*) [48–50]. The remaining species are categorized as “vulnerable”—the great one-horned rhinoceros (*Rhinoceros unicornis*) [51]—and “near threatened”—the white rhinoceros [52].

The primary drivers of this decline include habitat loss and fragmentation, along with widespread poaching [53]. This leads to thinner, fragmented populations that lose genetic diversity, pushing rhinoceros species closer to extinction. The threat extends beyond the existence value of the Rhinocerotidae family. As megaherbivores, rhinoceroses serve as crucial ecosystem architects [54], and their decline can result in significant ecological degradation. Moreover, rhinoceroses are charismatic species [55] that provide essential cultural services and serve as prominent symbols for conservation efforts. Their presence is vital for promoting sustainable ecotourism, benefiting both biodiversity conservation and local communities. The extinction of one or more rhinoceros species would result in significant losses across the diverse values associated with biodiversity, encompassing both ecological significance and human relationships [56–58].

There appears to be awareness among the three national samples regarding the crisis rhinoceros are facing. A significant proportion of respondents correctly identified the black rhinoceros as critically endangered, with poaching recognized as the primary threat. However, a concerning trend emerging from responses to Q5 is the underestimation of political and social unrest as threat factors. This suggests a tendency to view poaching in isolation, detached from the broader contexts that facilitates or drives it. Such a perspective can lead to a superficial understanding of the crisis and an underestimation of its underlying causes.

### 4.3. Communication, media, and awareness

National engagement in rhinoceros conservation and the visibility of related initiatives may contribute to shaping public awareness. Q4, in this regard, shows that Czech respondents are particularly adept at assigning the correct critical threat level to black rhinoceroses, suggesting a greater awareness on the rhinoceros crisis. This distinctive aspect of the Czech sample correlates with higher media exposure compared to the other two countries included in the study (**Fig 2**).

Logistic models suggest that this high media coverage in Czechia does not influence support for ART or other new conservation approaches, nor does it affect indecision when assessing a species’ threat level. Further research is needed to determine instead whether media coverage specifically enhances the public’s ability to identify the threat status of black rhinoceros. Nonetheless, a strong likelihood exists for a connection between the two. Media have a strong influence on shaping conservation narratives [59] and rhinoceroses have achieved an icons status in Czechia through sustained communication and outreach efforts in recent years.

The breeding of rhinoceroses, representing a success story over the past fifty years at Dvůr Králové Zoo in Czechia, is a popular topic, bolstered by the general popularity of zoos across the country (in 2021, six of the ten most visited tourist attractions were zoos), which broadens the reach of conservation messaging. The successes in breeding and conservation efforts have fostered a sense of national pride, gradually becoming news that Czech media actively cover, reflecting gatekeeping practices in selecting stories for public interest. Consequently, even minor successes or routine events are frequently reported in Czech media, such as rhinoceros horn burnings, horn trimmings, or short distance transportation.

Moreover, rhinoceroses have become iconic animals in Czechia, as demonstrated by notable events such as the installation of a stone statue of Sudan, the last NWR, at a roundabout in Dvůr Králové, and an exhibit of its dermoplastic remains and skeleton at the National Museum in Prague.

This demonstrates the potential impact of sustained, focused communication efforts by zoos and conservation institutions on public environmental awareness. Through a long-term commitment to education and media engagement, these institutions can cultivate a positive media climate for conservation-related news, creating a public that is not only well-informed but also actively supportive of conservation.

### 4.4. Low-tech and hi-tech approaches to conservation

Responses to Q7 and item 10 of Q8 indicate that respondents generally prefer “traditional” and “low-tech” conservation methods over newer, “hi-tech” approaches, such as ART and GRB (**Table 4** and **5**). An exception to this trend involves zoos and aquaria, which are the least preferred conservation strategies among Italian respondents and are also unpopular with the German sample, though they are generally liked by the Czech sample. Czech respondents are also those on average better disposed toward more innovative approaches.

A positive disposition for innovative solutions is associated with high levels of Prometheanism and a good disposition for environmental concern for biospheric reasons. Not surprisingly, individuals with strong confidence in humanity’s ability to address environmental challenges through technology are more inclined to support these advanced approaches. More striking is instead the fact that individuals who prioritize environmental issues from a broader, not exclusively human-centered perspective, are more open to solutions that the general public might find less appealing possibly because deemed “unnatural”, “invasive”, or simply “less traditional”. This willingness to consider all available means may indicate a more assertive attitude toward addressing the ecological and biodiversity crisis, where recognizing the gravity of the crisis aligns with a readiness to use every possible tool. This stance contrasts with a typical portrayal of non-anthropocentric environmentalism as inherently skeptical of technological and proactive solutions.

A preference for traditional methods over innovative ones is also reflected in responses to item 10 of Q7 (**Table 5**), though the public appears to recognize the importance of ART development for wildlife conservation (item 1, Q7). This likely suggests that the public does not, appropriately, view the two approaches as a dichotomy. Traditional and innovative methods should be seen not as competitors but as complementary strategies. This aspect still deserves to be further emphasized in communication. Protecting natural habitats remains the primary response to the threat of extinction, as no technological solution can fully replace this fundamental approach. However, technological methods can provide “safety nets” when traditional strategies fall short in mitigating specific threats. In the case of the NWR, for instance, the problem is not a lack of habitat, as plenty remain. Rather, the species has gone extinct in the wild due to poaching. The severity of the current biodiversity crisis makes it unrealistic to assume that functional populations of many endangered species can be sustained solely through traditional approaches. For many species, ART and GRB can serve as a last refuge, allowing them to “wait out” unfavorable conditions in the hope of more promising times.

### 4.5. The acceptance of ART in conservation

Despite the preference for more traditional conservation approaches, respondents still show a high level of acceptance for the use of ART in conservation (**Tables 4** and **5**). This is a positive finding, especially considering the skepticism that often surrounds many biotechnologies—particularly those applied to plants and animals. While there has been considerable public support for biotechnologies in human health (so-called “red biotechnologies”), public opinion surveys have shown much lower acceptance for their application in agriculture (“green biotechnologies”) [39].

The acceptance of ART for conservation could likely be increased even more by stressing some strategic points. First, the purpose of ART use is crucial: wildlife conservation holds significance for a vast array of reasons. This fact may resonate more clearly and easily with those who hold biospheric concerns. Indeed, the logistic model shows that one factor that seems to increase acceptance of ART in conservation is a stronger emphasis on biospheric motivations for environmental protection. In this way, it may be helpful to invest more in communicating the anthropocentric reasons for wildlife conservation so that the importance of ART and other advanced conservation methods can become more apparent also to those with less biospheric inclinations.

One possible approach here could be to emphasize the role of ART in supporting ecosystem health, which in turn is fundamental to human well-being. Framing ART as interventions in animal reproductive health—an essential component of ecosystem health—would align with the One Health approach advocated by the FAO, World Health Organization, World Organization for Animal Health and the United Nations Environmental Program [60]. The One Health approach underscores the interdependence between ecological, human and animal health. Using ART in conservation directly supports this interconnected health model by promoting environmental health through animal health, thereby creating benefits for people as well.

Another factor supporting the acceptance of ART in conservation is the public familiarity with these techniques, many of which were originally developed and are routinely used in human medicine. Highlighting this fact in communications can help normalize ART applications in conservation, making their use seem more intuitive and acceptable in a broader environmental context.

### 4.6. The importance of ethical reflection on ART

One key issue emerging from the results is the need to pair the development of new strategies for conservation with appropriate ethical research. This concern is notably evident among the German sample (**Table 5**), and may partly explain why German respondents express slightly more caution about ART compared to Czech and Italian respondents.

The significance of ethical analysis in this context cannot be overstated. Without sustained external intervention, debates on new or not well established technologies risk being dominated by unfounded fears or alarmist rhetoric, ultimately eclipsing reasoned, evidence-based arguments [61]. A classic example were the public concerns over cloning following the announcement of Dolly the sheep. Without structured ethical analysis to guide public discourse, discussions can easily drift toward irrational concerns, potentially delaying or undermining the acceptance of beneficial biotechnologies and, in this case, leading to the preventable loss of further biodiversity.

This also means that legitimate ethical concerns must be timely addressed. Wildlife conservation ethics intersects three major value domains: environmental ethics, animal ethics, and social ethics [62]. Balancing the moral imperatives from each of these domains presents a significant challenge. Concerning the application of ART to conservation, two primary concerns arise: efficient resource use and ensuring animal welfare.

On the first point, both Italian and German respondents express a degree of pessimism about the possibility of saving species with very small populations (in line, as far as the Italian sample is concerned, with the fatalism that emerged from the responses to Q3). In contrast, Czech respondents display more optimism, potentially due to a slightly higher level of Prometheanism within the sample. Nevertheless, all groups are open to funding ART research as a last-resort measure to save critically endangered species.

Regarding animal welfare, all three samples agree that while individual animals may contribute to species conservation, efforts should be made to not compromise their well-being. These concerns underscore the importance of conducting rigorous risk assessments before ART applications, along with ongoing monitoring of animal health throughout the process [26]. This focus on animal welfare is increasingly prevalent in conservation, as ignoring welfare concerns, besides being a morally controversial conduct, could erode societal support for conservation initiatives [63]. Moreover, in cases where breeding and reintroduction programs are essential, especially for species with limited populations, the goals of conservation and animal welfare tend to align closely [64,65].

One might wonder how one should proceed if a substantial part, or even the majority, of the population were opposed to the use of ART. Such opposition does not in itself imply that applying ART in conservation would be wrong, but it does indicate the need for greater attention to the conditions under which these techniques can be used responsibly. Clear communication becomes important to explain what the techniques involve, why they are being considered, and what their expected benefits and limitations are, so that decisions are not perceived as opaque or overly technocratic. Providing opportunities for consultation can also help ensure that concerns or questions from relevant groups are heard and, where appropriate, taken into account in shaping how the techniques are applied. Whether these measures would be sufficient depends on several contextual factors. In any case, this situation does not appear to arise in the national samples examined here: attention to the ethical issues associated with the use of ART in conservation does not translate into their rejection.

### 4.7. Limitations and future developments

Several key findings emerged in this research concerning public perception and knowledge regarding the environmental crisis, the rhinoceros crisis, and the application of ART in conservation. The main limitation of this study relate to the representativeness of the selected countries. While each sample is appropriately representative in number and composition for its respective nation, the study’s geographic scope is limited. The three countries selected are illustrative of Europe’s central and southern regions and of the three primary European language families. However, to fully capture Europe’s diverse cultural perspectives, future research should include additional samples from Western, Northern, and Eastern Europe. Moreover, conducting comparative studies on other continents could provide a more comprehensive view of public opinion across diverse cultural contexts.

Another limitation lies in the media analysis, which focused exclusively on newspapers. Although newspapers are accessible and influential media sources, they only cover a portion of society’s complex information landscape. Moreover, the relationship between media coverage and public awareness of the rhinoceros crisis could be further examined.

Finally, this study’s focus was limited to classical ART, although aART with potential conservation applications are emerging. There is, in this sense, a need for future research on techniques such as stem cell–derived in vitro gametogenesis or gene-editing methods when applied to wildlife conservation. It is, in fact, unclear whether these approaches would receive the same level of public acceptance as classical ART. They are less well-established, less familiar, and less easily understood by the general public. Moreover, they have the potential to redefine in more radical ways the concepts of reproduction and extinction, introduce additional technological elements to conservation, and further blur the boundaries of the common-sense distinction between natural and artificial. Consequently, we should not assume that the hopes, concerns, and expectations that the public holds regarding classical ART in wildlife conservation would apply equally to aART, especially given their potentially different ethical and conceptual ramifications, highlighting the importance of continued research on public perception of these emerging techniques.

## 5. Conclusions and recommendations

In this study, we investigated public perceptions in three European countries—Czechia, Germany, and Italy—regarding the application of classical ART to wildlife conservation, alongside respondents’ attitudes towards the ecological crisis as well as their knowledge about the biodiversity and rhinoceros crises. The results suggest several possible steps to improve communication about these topics and foster acceptance of ART into conservation.

There is a good level of public acceptance of application of classical ART in wildlife conservation. However, the results suggest improving awareness amongst the European public of the threat levels facing European species, which tend to be underestimated compared to exotic species. Further, there is a need to highlight the contributions of social and political factors to extinction risks and to promote conservation-related communication led by zoos and other ex-situ conservation institutions, which can help bring these topics into mainstream media. As popularity of zoos and aquaria seem to be waning in countries like Germany and Italy, activism towards conservation can become important not only to defend biodiversity but also to restore the popularity of these institutions.

To encourage a more widespread positive attitude toward ART and other innovative conservation approaches, communication efforts should emphasize the complementarity of these biotechnologies with conventional conservation methods rather than presenting them as mutually exclusive. Since individuals who are motivated primarily by anthropocentric reasons to protect the environment tend to be more skeptical of these approaches, communication should highlight the benefits that conservation strategies, including ART, offer to humanity and future generations. One effective strategy is to integrate these approaches within the One Health framework, which considers ecosystem health, animal health, and human health as interconnected dimensions.

Finally, our findings confirm the importance of accompanying ART research and development in conservation with a careful ethical analysis. This approach would ensure the responsible implementation of these biotechnologies and align with public expectations for ethical oversight.

## Supporting information

S1 File Survey, English version. (PDF)

S2 File Demographics. (PDF)

S3 File Media analysis newspapers. (PDF)

S4 File Models. (PDF)
