# Supplementary material for: Perceptions of assisted reproductive technologies in wildlife conservation: Public expectations and ethical implications across three EU countries
Source: PLoS One. 2026 Feb 27;21(2):e0342094. doi: 10.1371/journal.pone.0342094 (PMC12948088; doi:10.1371/journal.pone.0342094)
Supplement: S1 File — (PDF) [file pone.0342094.s001.pdf]

Perceptions of Assisted Reproductive Technologies in Wildlife Conservation: Public Expectations and Ethical Implications Across Three EU Countries

Supplementary material 1

Survey (English version—distributed surveys were translation of this questions in Czech, German and Italian)

Demographics

D1—Gender
• Male
• Female
• Other
• Prefer not to answer

D2—Age:

D3—Level of education
What is your highest level of completed education?
• None or primary school
• Secondary school up to the mandatory education age
• High school diploma (beyond the mandatory education age)
• University*

D3*—Degree Field
In which field did you graduate?
• Law and Economics
• Life and Environmental Sciences (e.g., Biology, Forestry, Natural Sciences, Environmental Sciences, Geology)
• Social Sciences (e.g., Psychology, Sociology, Education)
• Humanities (e.g., Literature, History, Philosophy)
• Sciences, Mathematics, Physics, and Engineering (e.g., Chemistry, Mathematics, Physics, Technology, Engineering)
• Medicine and Healthcare Professions
• Veterinary Medicine

D4—Region:

D5—Province:

D6—Municipality:

D7—Current Job
What is your current occupation?
• Manual worker

- Technician, employee, or officer
- Executive
- Merchant
- Artisan
- Freelancer (e.g., lawyer, doctor, surveyor, etc.)
- Entrepreneur
- Student
- Homemaker
- Unemployed
- Retired
- Armed forces or voluntary civil service
- Other

D8—Sector
In which sector do you work or have worked?
- Public
- Private

Questionnaire

Q1—People generally worry about environmental issues because of their consequences. However, these consequences can be of different kinds. Please rate each of the following items from 1 (not important) to 7 (supreme importance):
I am concerned about environmental issues because of the consequences for:
- Plants
- Animals
- My health
- My future
- All people
- Future generations

Q2—How often have you done each of the following action in the past year? (alternatives: Never; Rarely; Sometimes; Often)
- Looked for ways to reuse things.
- Recycled newspapers, cans or bottles.
- Encouraged friends or family to recycle.
- Purchased products in reusable containers.
- Picked up litter that was not my own.
- Composted food scraps.
- Avoided to use gasoline by walking or bicycling.
- Wrote a letter supporting an environmental issue.
- Voted for a candidate who supported environmental issues.
- Donated money to an environmental group.
- Volunteered time to help an environmental group.

Q3—Regarding the environment could you please tell us how much you agree (Totally disagree; Mildly disagree; Mildly Agree; Totally Agree; Don't know) with the following statements:
- We are approaching the maximum number of people that Earth can support.
- The Earth is like a spaceship with very limited room and resources.
- Humans have the right to modify Nature to suit their needs.
- Human ingenuity will ensure that we do not make the Earth unlivable.
- The balance of Nature is strong enough to cope with the impacts of modern industrial nations.
- The so-called ecological crisis facing humankind has been greatly exaggerated.
- Humans were meant to rule over the rest of Nature.
- When humans interfere with the environment it often produces disastrous consequences.
- Humans are severely abusing the environment.

- Despite our special abilities, humans are still subject to the laws of ecology.
- If things continue on their present course, we will soon experience a major ecological catastrophe.

Q4—The current rate of wildlife extinction, according to scientists, is accelerating dramatically. Focusing on mammals, could you assess the extinction risk of the following species based on your knowledge? Put a score from 1 (not endangered) to 7 (highly in danger) for each species:

- Black rhino (*Diceros bicornis*)
- Lion (*Panthera leo*)
- Black wildebeest (*Connocaetes gnou*)
- European mink (*Musteoloa lutreola*) [For Italy: Mediterranean monk seal (*Monachus monachus*)]
- Brown bear (*Ursus arctos*)
- European roe deer (*Capreolus capreolus*)

Q5—All the extant rhino species are a symbol of the biodiversity crisis. To the best of your knowledge, what are the worst threats to their survival? Put a score from 1 (not threatening) to 7 (highly threatening) for each threat:

- Invasive species
- Poaching
- Competition with other species
- Habitat loss
- Predators
- Pollution
- Political and social unrest
- Natural disasters

Q6—In an attempt to contrast biodiversity loss, several public-funded projects make efforts to save endangered species. The BioRescue project is devoted to saving the Northern white rhino from extinction. The Northern white rhino is extinct in the wild, and the captive population is composed of just two females, the last male having died in 2018. Projects like BioRescue work for developing and applying advanced assisted reproductive technologies (e.g., in vitro fertilization) to preserve wild species from extinction. Please rate each of the following items from 1 (less acceptable) to 7 (most acceptable).

- Governments should invest public money to support research into advanced assisted reproductive technologies for conserving species extinct in nature.
- Saving a single animal of a species is a first step toward the conservation of the species itself.
- Conservation of almost extinct species is pointless.
- Research groups applying advanced assisted reproductive technologies to protect wild species from extinction should be ethically regulated by the government that funds them.
- We should not push to the point of putting at risk the individual animals, even in trying to save a species.
- Individual animals can be means to reverse the extinction of their species.
- Advanced assisted reproductive technologies should be funded to attempt to preserve almost extinct species.
- Research groups into advanced assisted reproductive technologies for conservation of biodiversity funded by the government should carry out their research activities freely with no ethical constraints.
- Governments should invest public money to support projects which aim to protect endangered species or sub-species rather than those already extinct in nature.
- Governments should support projects which aim to protect natural habitats and limit pollution rather than using advanced reproductive technologies for conserving biodiversity.

Q7—How do you rate the following approaches to biodiversity conservation on a scale from 1 (unacceptable) to 7 (acceptable)?

- Zoos and aquariums
- Creation of protected areas
- Captive breeding and re-introduction for animals
- Assisted reproduction for animals
- Seed banks
- Botanical gardens

- Biobanking (i.e. cryopreservation of genetic samples)

Q8—Of the following list of sources of information about developments in science and technology, please choose the two main sources that you use (watch, read, or listen) the most.
- Television, on a TV set or via the internet
- Newspapers, either online or in print
- Online encyclopedias (e.g. Wikipedia)
- Magazines, either online or in print
- Radio, including podcasts
- Books, either in print or e-books
- Online social networks and blogs (e.g. video hosting websites)
- Scientific journals, either online or in print
- Other (SPONTANEOUS)
- You do not look for information about developments in science and technology
- Do not know
