# Supplementary material for: Perceptions of assisted reproductive technologies in wildlife conservation: Public expectations and ethical implications across three EU countries
Source: PLoS One. 2026 Feb 27;21(2):e0342094. doi: 10.1371/journal.pone.0342094 (PMC12948088; doi:10.1371/journal.pone.0342094)
Supplement: S2 File — (PDF) [file pone.0342094.s002.pdf]

Perceptions of Assisted Reproductive Technologies in Wildlife Conservation: Public Expectations and Ethical Implications Across Three EU Countries

Supplementary material 2

Demographics (weighted percentages)

		**Czech Republik (n=449)**		**Germany (n=877)**		**Italy (n=737)**	
Age classes	Education attainment (International Standard Classification of Education - ISCED)	Male	Female	Male	Female	Male	Female
Y20-24	Less than primary, primary and lower secondary education (levels 0-2)	0,46%	0,34%	1,69%	1,36%	0,90%	0,56%
	Upper secondary and post-secondary non-tertiary education (levels 3 and 4)	3,45%	3,11%	3,16%	3,02%	3,68%	3,51%
	Tertiary education (levels 5-8)	0,36%	0,58%	0,48%	0,63%	0,49%	0,69%
Y25-34	Less than primary, primary and lower secondary education (levels 0-2)	0,83%	0,91%	2,23%	1,74%	2,66%	1,96%
	Upper secondary and post-secondary non-tertiary education (levels 3 and 4)	7,48%	5,26%	5,82%	5,23%	5,57%	4,62%
	Tertiary education (levels 5-8)	3,02%	4,55%	4,32%	4,55%	2,48%	3,62%
Y35-44	Less than primary, primary and lower secondary education (levels 0-2)	0,67%	0,61%	2,14%	1,89%	2,97%	3,09%
	Upper secondary and post-secondary non-tertiary education (levels 3 and 4)	9,35%	7,56%	5,62%	5,78%	5,28%	5,34%
	Tertiary education (levels 5-8)	3,87%	4,70%	4,35%	4,15%	2,66%	3,79%
Y45-54	Less than primary, primary and lower secondary education (levels 0-2)	0,55%	0,64%	2,16%	2,25%	6,77%	5,83%
	Upper secondary and post-secondary non-tertiary education (levels 3 and 4)	10,82%	9,73%	6,32%	7,06%	6,47%	6,94%
	Tertiary education (levels 5-8)	2,86%	5,87%	4,23%	3,32%	2,47%	3,21%

Y55-64	Less than primary, primary and lower secondary education (levels 0-2)	0,20%	0,55%	1,24%	1,45%	3,26%	3,05%
	Upper secondary and post-secondary non-tertiary education (levels 3 and 4)	5,51%	3,20%	5,12%	5,08%	2,42%	3,78%
	Tertiary education (levels 5-8)	1,30%	1,67%	1,86%	1,77%	0,85%	1,06%
Total		50,73%	49,27%	50,74%	49,26%	48,95%	51,05%
