# Supplementary material for: Perceptions of assisted reproductive technologies in wildlife conservation: Public expectations and ethical implications across three EU countries
Source: PLoS One. 2026 Feb 27;21(2):e0342094. doi: 10.1371/journal.pone.0342094 (PMC12948088; doi:10.1371/journal.pone.0342094)
Supplement: S3 File — (PDF) [file pone.0342094.s003.pdf]

# Perceptions of Assisted Reproductive Technologies in Wildlife Conservation: Public Expectations and Ethical Implications Across Three EU Countries

## Supplementary material 3

### Media Analysis Newspapers

| Country | Query | Newpapers | Archive |
|---|---|---|---|
| ***Czechia*** | *Nosorožec tuponosý* | denik.cz<br>novinky.cz | Websites of the newspapers |
| ***Germany*** | *Breitmaulnashorn* | suddeustche.de<br>bild.de | Lexis Uni |
| ***Italy*** | *Rinoceronte bianco* | corriere.it<br>repubblica.it | TIPS project |
