# Supplementary material for: Perceptions of assisted reproductive technologies in wildlife conservation: Public expectations and ethical implications across three EU countries
Source: PLoS One. 2026 Feb 27;21(2):e0342094. doi: 10.1371/journal.pone.0342094 (PMC12948088; doi:10.1371/journal.pone.0342094)
Supplement: S4 File — (PDF) [file pone.0342094.s004.pdf]

Perceptions of Assisted Reproductive Technologies in Wildlife Conservation: Public Expectations and Ethical Implications Across Three EU Countries

Supplementary material 4

Model 1: ART Support (*significant; ** very significant)

Model	Deviance	AIC	BIC	df	X^2	p	Nagelkerke R^2
H_0	1745.425	1747.425	1752.732	1489			
H_1	1489.580	1515.580	1591.871	1477	255.845	< .001	0.229

Coefficients

					Wald Test	95% Confidence interval	
	**Estimate**	**Standard Error**	**Odds Ratio**	**z**	**p**	**Lower bound**	**Upper bound**
(Intercept)	-0.929	0.324	0.395	-2.868	0.004	-1.564	-0.294
Concerned about biosphere**	1.367	0.146	3.925	9.365	< .001	1.081	1.654
Prometheanism	0.019	0.162	1.019	0.117	0.907	-0.299	0.337
Limits to growth*	0.454	0.144	1.575	3.155	0.002	0.172	0.737
Fatalism**	0.800	0.147	2.226	5.429	< .001	0.511	1.089
Education: Upper secondary and post-secondary non-tertiary education	0.149	0.161	1.161	0.929	0.353	-0.166	0.464
Education: Tertiary education	-0.028	0.186	0.972	-0.153	0.878	-0.392	0.335
Age: 25 - 34 years old	0.314	0.240	1.369	1.311	0.190	-0.156	0.783
Age 35 - 44 years old*	0.470	0.240	1.600	1.962	0.050	0.000	0.940
Age 45 - 54 years old	0.263	0.232	1.300	1.131	0.258	-0.192	0.717
Age 55 - 64 years old	0.423	0.239	1.527	1.774	0.076	-0.044	0.891
Gender (Male)	-0.093	0.131	0.911	-709	0.478	-0.350	0.164
Country (de)*	-0.419	0.154	0.666	-2.720	0.007	-0.720	-0.117
Media coverage (High)	0.146	0.185	1.157	0.789	0.430	-0.216	0.508

Note. ART Support level '1' coded as class 1. Model performance: Accuracy= 0.777; F-measure=0.859

Model 2: Support for high tech strategies for biodiversity conservation (*significant; ** very significant)

Model	Deviance	AIC	BIC	df	X^2	p	Nagelkerke R^2
H_0	967.057	969.057	974.358	1481			
H_1	929.798	957.798	1039.315	1468	37.259	< .001	0.052

Coefficients

					Wald Test	95% Confidence interval	
	Estimate	Standard Error	Odds Ratio	z	p	Lower bound	Upper bound
(Intercept)	0.624	0.418	1.866	1.492	0.136	-0.196	1.444
Concerned about biosphere*	0.584	0.208	1.794	2.814	0.005	0.177	0.991
Prometheanism **	0.646	0.204	1.909	3.168	0.002	0.246	1.046
Limits to growth	0.325	0.201	1.384	1.619	0.105	-0.068	0.719
Fatalism	0.268	0.216	1.307	1.236	0.216	-0.157	0.692
Education: Upper secondary and post-secondary non-tertiary education	0.078	0.213	1.081	0.365	0.715	-0.340	0.496
Education: Tertiary education	0.245	0.259	1.277	0.944	0.345	-0.264	0.753
Age: 25 - 34 years old	0.037	0.319	1.038	0.116	0.908	-0.588	0.662
Age 35 - 44 years old	0.240	0.324	1.271	0.741	0.459	-0.395	0.875
Age 45 - 54 years old	0.193	0.313	1.213	0.616	0.538	-0.421	0.807
Age 55 - 64 years old	0.313	0.325	1.367	0.964	0.335	-0.323	0.949
Gender (Male)	0.282	0.178	1.325	1.584	0.113	-0.067	0.630
Country (de)	-0.228	0.208	0.796	-1.099	0.272	-0.636	0.179
Media coverage (High)	0.323	0.271	1.381	1.192	0.233	-0.208	0.853

Note. Support High Technological strategies for Biodiversity conservation level '1' coded as class 1. Model performance: Accuracy= 0.899 F-measure=0.947

Model 3: Being in doubt about endangered species

Model	Deviance	AIC	BIC	df	X^2	p	Nagelkerke R^2
H_0	1.811.823	1.813.823	1.819.129	1489			
H_1	1.739.505	1.765.505	1.841.797	1477	72.317	< .001	0.067

Coefficients

					Wald Test	95% Confidence interval	
	Estimate	Standard Error	Odds Ratio	z	p	Lower bound	Upper bound
(Intercept)	1.329	0.321	3.777	4.139	< .001	2.013	7.086
Concerned about biosphere	-0.069	0.157	0.933	-0.441	0.641	0.687	1.269
Prometheanism**	0.547	0.133	1.727	4.119	< .001	1.332	2.241
Limits to growth	0.145	0.130	1.156	1.117	0.220	0.896	1.491
Fatalism	-0.254	0.149	0.775	-1.707	0.078	0.579	1.038
Education: Upper secondary and post-secondary non-tertiary education	-0.063	0.149	0.939	-0.423	0.835	0.702	1.257
Education: Tertiary education	-0.070	0.172	0.932	-0.407	0.674	0.665	1.306
Age: 25 - 34 years old	-0.218	0.261	0.804	-0.834	0.641	0.482	1.342
Age 35 - 44 years old	-0.497	0.253	0.608	-1.964	0.451	0.370	0.999
Age 45 - 54 years old	-0.766	0.245	0.465	-3.122	0.056	0.287	0.752
Age 55 - 64 years old**	-1.051	0.247	0.350	-4.256	0.002	0.216	0.567
Gender (Male)**	-0.023	0.118	0.977	-0.197	< .001	0.775	1.232
Country (de)	0.037	0.164	1.038	0.228	0.841	0.753	1.431
Country (it)	-0.043	0.138	0.958	-0.312	0.178	0.731	1.255
Media coverage (High)	0.037	0.164	1.038	0.228	0.819	0.753	1.431

Note. Being in doubt about endangered species level '1' coded as class 1. Model performance: Accuracy= 0.702 F-measure=0.820
